# The Modern Treatment of Ophthalmia Neonatorum

**Published:** 1908-02-29

**Authors:** Rosa Ford

**Affiliations:** Clinical Assistant to the Royal Eye Hospital, Southwark, London.


### THE MODERN TREATMENT OF OPHTHALMIA NEONATORUM.

By ROSA FORD, M.B.(Lond.), Clinical Assistant to the Royal Eye Hospital, Southwark, London.

Although expert advice is always desirable in every case that is not evidently quite mild, there must always be babies whom the general practitioner is obliged to treat himself, and a knowledge of the results obtained by careful experiments may therefore be of value to him. The following detail of treatment is one that gives very satisfactory results:—

*Prophylaxis.*—(i) Wipe the eyelids clean with a *damp* swab immediately after birth. (ii.) Drop protargol 10 per cent. into each conjunctival sac as soon as possible after birth.

*Treatment.*—(i) Cleanse the conjunctival sac every half-hour with a warm solution of borax (ʒij. ad. Oj.). (ii.) Foment the eyes with squares of gauze wrung out of a hot solution of the same kind. Change every half-hour. Do not cover the gauze with anything else. (iii.) Drop protargol 10 per cent. into the conjunctival sac every hour. (iv.) Once a day instil protargol 20 per cent., keeping the solution in the conjunctival sac for two minutes.

*If the cornea becomes hazy.*—Instil atropine (grs. ij. ad ℥j.) three times a day, and continue all the other measures.

*In hyper-acute cases and in relapses.*—Treat as above until the tense swelling is reduced and the secretion free, then make a single alteration; instead of instilling the 20 per cent. protargol, paint the whole of the palpebral conjunctiva with 2 per cent. silver-nitrate solution once a day. After a minute, wash off any excess of fluid with sterilised salt solution.

### Remarks on Prophylactic Measures.

A damp swab for cleansing the eyelids is preferable to a dry one, or to dry rags, because these stick to the greasy skin surface, and are thus more difficult to manipulate, and less certain to remove all trace of foreign matter. Wet swabs are also inadvisable, since the cleansing solution, now charged with infected matter, will in all probability leak into the conjunctival sac, and thus produce the very evil it is desired to avert. For the same reason damp swabs, *i.e.* swabs wrung out of boiled water as "dry" as possible, should be prepared shortly before birth, and should lie to hand in a clean, dry vessel.

It is not now necessary to advocate the use of Crédé's method of prophylaxis. It is common knowledge that the percentage of cases of ophthalmia in maternity hospitals, formerly 4 to 19 per cent. in different institutions, has been reduced by its use to 0.2 per cent. But the objection in private practice is the inflammation which it causes. This cannot be remedied by using a weaker silver nitrate solution than 2 per cent., for this diminishes the protective effect. Neither is perchloride of mercury a good substitute, since this, in the strength required, also causes irritation. v. Herff, however, has demonstrated that protargol is an efficient prophylactic. In 3,000 children in the Basle Women's Hospital who were treated in this manner not a single case of ophthalmia occurred. This protein silver salt causes only a slight irritation, lasting but a few minutes.

### Remarks on Therapeutic Measures.

The object of the cleansing solution is simply to remove dead infected matter as readily as possible, and this is best effected by a lotion which is bland, alkaline, and antiseptic. Borax possesses all these qualities, and it is also readily soluble in water. Although perchloride of mercury is a stronger antiseptic, yet it is irritant, and its frequent use tends to increase inflammation.

Warm fomentations are preferable to ice compresses, because they encourage a free circulation, thus enabling the leucocytes to make a better fight against the micro-organisms. In all probability the fomentations have no effect on the organisms themselves. Ice compresses may certainly retard the activity of the bacilli, but they contract the vessels, hindering the circulation and thus also the process of cure. They also depress the vitality of the tissues, and so favour keratitis and ulceration of the cornea. Gauze exerts only a light pressure on the eyelids, and is kept moist by constant changing; this moisture prevents the secretion from becoming dry, sealing the lids together and damming back the pus. Vaseline or ointment is therefore unnecessary, and it is an advantage to be able to dispense with both, since their use makes the lids slippery and examination or treatment difficult.

### Silver Preparations Contrasted.

Thus far effects only have been dealt with. To combat the causative bacilli three drugs present themselves for choice—namely, nitrate of silver, protargol, and argyrol. The actions of the two latter drugs have been widely studied of late, and opinions differ greatly as to their value. That protargol is the most valuable of the three is shown by the results obtained by the following observers:—

Harrison Butler ("Ophthalmoscope," January 1907, p. 14) compared the effects of the three salts by using a different one for each eye in the same individuals. His cases were mostly caused by the Koch-Weeks bacillus, and he concludes that protargol (33 per cent.) is slightly better than silver nitrate (2 per cent.), and markedly better than argyrol (33 per cent.), while of the last two the latter is slightly the superior.

Myles Standish ("Ophthalmic Record," 1906, p. 395) compared the effects of these three drugs in cases of ophthalmia neonatorum while the cornea is still clear. His results are as follows:—

Of 50 cases treated with silver nitrate, 6 per cent. developed corneal mischief.

Of 150 cases treated with protargol, 2 per cent. developed corneal mischief.

Of 201 cases treated with argyrol, 2 per cent. developed corneal mischief.

Marshall and Neave ("British Medical Journal," August 18, 1906, p. 359), by a series of experiments undertaken at the request of the Therapeutical Committee of the British Medical Association, proved in the laboratory that silver nitrate and protargol are equal in antiseptic power, while argyrol has very little such action. All three salts were used with the same percentage of silver, but argyrol, even in much greater strength, still shows but slight antiseptic properties.

v. Herff ("Münchener Med. Wchnschr.," 1906, p. 958), by laboratory experiments with protargol and argyrol, obtained the same results.

Clinically, then, protargol and argyrol are more useful than silver nitrate, and of the two protargol is the more efficacious. Laboratory experiments indicate protargol and silver nitrate as most active, and since the latter is less effective in practice the final choice falls upon the former.

### The Dangers of Silver Nitrate.

Silver nitrate is a dangerous drug. Darier makes the following bold statement: "Complications are generally caused by unduly strong solutions of silver nitrate." Baillart says that silver nitrate, if not very prudently used, may cause alteration of corneal epithelium; and this is the very evil we are anxious to avoid. Also silver nitrate is painful; protargol is comparatively painless. Silver nitrate should be used by the physician only; the application of protargol may be entrusted to the nurse or attendant. Silver nitrate must not be used in the early stage; protargol may be used throughout. Silver nitrate can only be used once a day; protargol may be instilled with great frequency.

In very acute cases, however, silver nitrate is the better drug. Baillart says that the gravest cases are never so rapidly improved by protargol as by silver nitrate. Darier also recommends its use in relapses, which are often hard to overcome.

### Practical Hints.

1. In cases of ante-partum ophthalmia, when the child is born with the eyes already inflamed, do not apply Credé's treatment. There is a tendency to think that if it is useful as a routine, much more is the method of value when inflammation is actually present. This is a mistake. The disease is already in its first stage, and silver-nitrate solution will only do harm by increasing inflammation.

2. If Credé's method has been neglected at birth, do not apply it on the first sign of inflammation. It is then too late.

3. Never use silver-nitrate solution stronger than 2 per cent. Mistakes are so often made, and the results are so disastrous, that it is well to emphasise the caution that a solution labelled 10 per cent. is 5 times as strong as one labelled 10 grains to the ounce (2 per cent.).

4. Examine the cornea of a child with ophthalmia neonatorum at least twice a day. There are only two ways of doing this. The child's hands should be securely held by one assistant, the head absolutely fixed by another, or between the operator's knees. Then the tips of the thumbs should be placed opposite one another on the extreme borders of the child's lids, and while exerting a gentle but firm pressure on the eyeball, and also holding the two thumbs constantly parallel to each other, the lids should be separated as far as possible. Maintain this position steadily until the child moves the eye, as it is sure to do sooner or later, so that the cornea comes for a brief moment into full view. The other method is by metal retractors, which should only be used as a last resort.

5. Protargol must be prepared with *cold* water. The best method is to dust the powder on to as large a surface as possible of the required amount of distilled water, and leave without stirring. It will dissolve in about half an hour. The solution does not keep well unless preserved in an amber-coloured bottle in a cool, dark place. A convenient form for the practitioner who does not often use it, is the *soloid* of 1 gr. or 4 grs.

6. If the inflammation is limited to one eye, it is in all probability a result of congenital duct obstruction. The case should be referred to an ophthalmic surgeon. Recognised early, these cases can be cured rapidly and easily, but if allowed to persist for some months, treatment is often difficult and tedious.

7. The percentage of cases of ophthalmia neonatorum due to the gonococcus has been estimated at 60 per cent. (Sydney Stephenson).

8. With regard to prognosis, De Schweinitz's opinion is valuable. He states that if an eye is seen while the cornea is still clear, except in diphtheritic or inherently malignant types, or in cases of malnutrition, a complete cure should be looked for. It has been estimated that 66 per cent. of the cases of ophthalmia neonatorum recover with unimpaired sight.

9. Protargol must not be used for too long. It undoubtedly stains the conjunctiva (Snell, de Schweinitz).

